# Understanding Water Equilibration Fundamentals as a Step for Rational Protein Crystallization

**DOI:** 10.1371/journal.pone.0001998

**Published:** 2008-04-23

**Authors:** Pedro M. Martins, Fernando Rocha, Ana M. Damas

**Affiliations:** 1 Grupo de Estrutura Molecular, Instituto de Biologia Molecular e Celular (IBMC), Universidade do Porto, Porto, Portugal; 2 Departamento de Engenharia Química, Faculdade de Engenharia, Universidade do Porto, Porto, Portugal; 3 Instituto de Ciências Biomédicas Abel Salazar (ICBAS), Universidade do Porto, Porto, Portugal; University of Washington, United States of America

## Abstract

**Background:**

Vapor diffusion is the most widely used technique for protein crystallization and the rate of water evaporation plays a key role on the quality of the crystals. Attempts have been made in the past to solve the mass transfer problem governing the evaporation process, either analytically or by employing numerical methods. Despite these efforts, the methods used for protein crystallization remain based on trial and error techniques rather than on fundamental principles.

**Methodology/Principal Findings:**

Here we present a new theoretical model which describes the hanging drop method as a function of the different variables that are known to influence the evaporation process. The model is extensively tested against experimental data published by other authors and considering different crystallizing conditions. Aspects responsible for the discrepancies between the existing theories and the measured evaporation kinetics are especially discussed; they include the characterization of vapor-liquid equilibrium, the role of mass transfer within the evaporating droplet, and the influence of the droplet-reservoir distance.

**Conclusions/Significance:**

The validation tests show that the proposed model can be used to predict the water evaporation rates under a wide range of experimental conditions used in the hanging drop vapor-diffusion method, with no parameter fitting or computational requirements. This model combined with protein solubility data is expected to become a useful tool for a priori screening of crystallization conditions.

## Introduction

Obtaining good quality crystals is a critical step for protein structure determination by X-ray crystallography. The most commonly employed techniques to grow crystals of biological macromolecules are by vapor diffusion [1]. In these techniques, the supersaturation state needed for crystallization to occur is achieved by slowly evaporating the solvent from a droplet containing the macromolecule buffered at a given pH, the crystallizing agent (or precipitant) and additives. Equilibration of the droplet takes place in a closed system, which also contains a reservoir with a solution at a higher precipitant concentration. Supersaturation, defined as the ratio between the macromolecule activity in solution and in a saturated state, increases during this process due to the solvent evaporation leading to the (i) increase of protein concentration and very often to (ii) its solubility decrease, due to the increasing concentrations of the precipitant. When the vapor pressure at the droplet surface equals the vapor pressure of the reservoir, equilibrium is attained and supersaturation is no longer affected by the solvent evaporation. The success of the method in obtaining well diffracting crystals is in a great deal determined by the kinetics of solvent evaporation and therefore by the numerous parameters governing the evaporation rate. If the process is too fast and supersaturation is built up to very high levels, the formation of an amorphous solid or a precipitate with bad diffracting qualities will take place; on the other hand, if evaporation takes place to a limited extent, situations may happen where no spontaneous nucleation will occur either because the solution is not yet supersaturated relatively to the macromolecule, or the supersaturation is not high enough to pass through the metastable region of no crystal formation [2].

**Figure 1:**
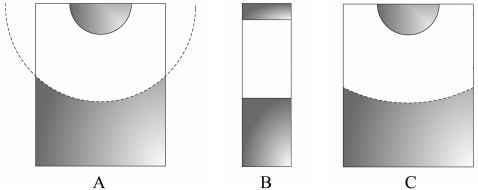
Schematic representation of the hanging drop method as considered in (A) Fowlis et al. [3], (B) Sibille et al. [4] and (C) Luft et al. [5] models. The interfacial area for mass transfer corresponds, respectively, to a spherical cap, to the cross-sectional area of a capillary tube and to an adjustable parameter of the model.

As a consequence of the key role of the solvent evaporation kinetics on the vapor diffusion technique, a comprehensive mathematical treatment was proposed for the first time in 1988 to describe the hanging drop method [3], in which the evaporating droplet is suspended over the reservoir due to its surface tension. The work was followed by two other approaches [4], [5] represented in Figure 1 by the respective schematic models. The Fowlis et al. model (FM) is based on the conventional arrangement of a Linbro box crystallization plate [3], while the formalism presented by Sibille et al. (SM) departs from different geometric constraints that reproduce capillaries closed at one end [4]. More recently, new equations were derived to describe vapor diffusion in an apparatus specifically designed for protein crystal growth in microgravity environment [6]. Identified limitations of the FM and SM to fit experimental data of water equilibration rates, led Luft et al. to propose an alternative model (LM) that can be viewed as a hybrid of the previous two models [5]. Nevertheless, the differences of the LM to its predecessors go beyond the geometric assumptions, since a non-physicochemical parameter is introduced – the effective surface area – that has to be evaluated by curve fitting of the LM to the observed evaporation kinetics. Empirical equations were previously proposed to describe the water equilibration rates measured in the presence of three widely used crystallization agents and combining the various experimental parameters [7]. The limitation of this type of equations is on their restricted applicability, which is confined to the set of conditions at which the empirical parameters were determined. In a different approach, numerical methods were employed to describe the equilibration of hanging drop experiments reported in literature; the software program “Drop” was presented as being based on the FM and SM but with fewer geometric assumptions, and was reported to adequately describe the experimental data [8].

The aim of this paper is to provide the mathematical description of the hanging drop vapor-diffusion method, so that it can be used to predict the water evaporation rates under a wide range of experimental conditions, without the limitations of the existing models. The new model shall be useful for obtaining good protein crystals regarding their quality, size and number, and should be combined in the future with complementary kinetic theories of crystal nucleation and growth [9]–[11].

## Analysis

**Figure 2:**
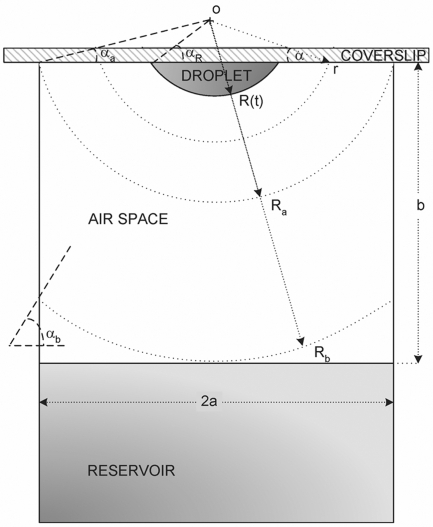
Scheme of the hanging drop method adopted on the derivation of the mathematical model.

As represented in Figure 2, the droplet is considered to be a spherical cap centered on the point O, with contact angle α*_R_*, and with a radius of curvature R that varies with time. The inner diameter of the reservoir is given by 2a, and the vertical distance from the point O to the cover slip corresponds to *R* sin α*_R_*. *R_a_* is the radius of the sphere centered on O that intercepts the reservoir walls at the level of the cover slide, and *R_b_* is the radius of the concentric sphere that is tangent to the surface of the solution in the reservoir:
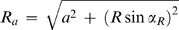
(1)

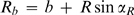
(2)When the droplet is a hemisphere, α*_R_* = 0°, and therefore *R_a_* = *a* and *R_b_* corresponds to the vertical distance from the cover slip to the solution in the reservoir, b. Different implications arise from the simplifying hypothesis adopted on the derivation of the model. This will be gradually illustrated by interpreting the limitations of the existing theories and by proposing new ways to overcome those limitations. We start by considering the simplest case of *R_a_*≥*R_b_*, and the evaporation kinetics is assumed to be exclusively determined by the solvent diffusion through the gas phase. Then, the more common case of *R_a_*<*R_b_* will be analyzed, and finally, the importance of the diffusion step within the droplet will be assessed. The model validation will be presented using experimental data reported in literature by different authors on the equilibration rates of different water-precipitant systems, under varied experimental conditions.

## Discussion

### Small droplet-to-reservoir distances (*R_a_*≥*R_b_*)

Let us start by considering the simplest case of small droplet-to-reservoir distances (*R_a_*≥*R_b_*) and that the evaporation of a droplet containing the solvent (water) and the precipitant occurs at a given rate that is exclusively determined by the vapor diffusion across the air space between the droplet and the reservoir. The latter assumption was initially justified in the derivation of the FM [3] and is common to the subsequent models, SM and LM [4], [5]. As the droplet with initial radius *R*_0_ gets progressively smaller, the geometrical center O moves toward the coverslip so as to provide that the droplet contact angle α*_R_* remains constant. This implies a small decrease of the radius *R_b_* (and *R_a_*) with time, which will be ignored since the droplet size is generally much smaller than b (and a) [3].

The mass transfer area (A) for vapor diffusion over the air space is of a spherical cap centered on O with radius r comprised between *R* and *R_b_*, and delimited by the coverslip. Accordingly,
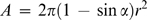
(3)When α*_R_*≠0°, the value of α becomes increasingly smaller than α*_R_* as one moves away from the droplet:
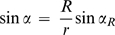
(4)The vapor pressure profile *p*(*r*) results from solving the continuity equation for steady-state:
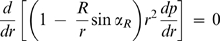
(5)subject to the boundary conditions *p*|*_r_*_ = *R*_ = *p_R_* and 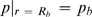:
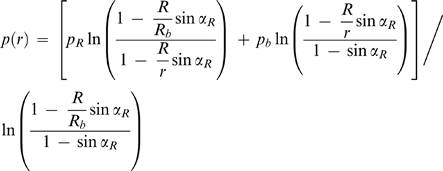
(6)In the mathematical treatment of Fowlis et al., the angle α is considered to be constant over the diffusion path [3]. As we have pointed out, this is only true for perfect hemisphere droplets. The solution found for the vapor pressure profile in the FM is therefore a limit case of Equation 6, when α*_R_* = 0°.

**Figure 3:**
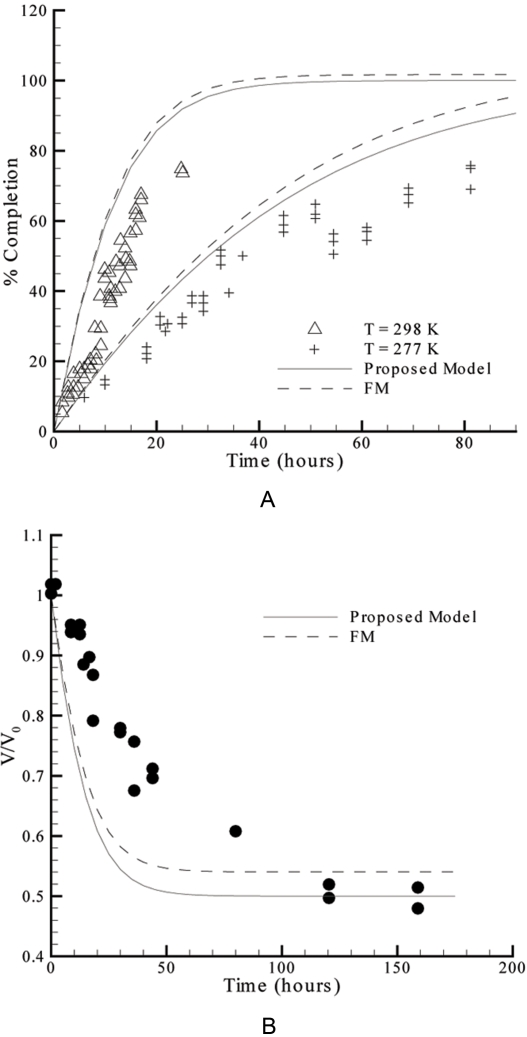
Plot of the theoretical water equilibration rates expected by the proposed model and by FM, and evaluation of the models against experimental data taken from literature. A: Experimental sets #1 (298 K) and #2 (277 K) of Table 1 [3]. B: Experimental set #3 of Table 1 [7].

The molar rate of water vapor leaving the droplet (*I*_1_) is evaluated at *r* = *R* according to Fick's first law:
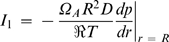
(7)where Ω*_A_* is the surface area shape factor of the droplet (2π(1−sinα*_R_*)), D is the diffusion coefficient of water vapor in air, ℜ is the gas constant and T is the absolute temperature. Hereafter, the subscript 1 stands for water and the subscript 2 for the crystallizing agent. Substituting Equation 6 into the previous equation, and letting
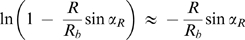
(8)one obtains that
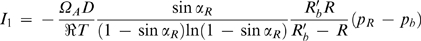
(9)where *R*′*_b_* reduces to *R_b_* when α*_R_* = 0°:
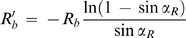
(10)The vapor pressure at the droplet surface *p_R_* decreases in the direct proportion to the water molar fraction in the droplet *x*_1_, following Raoult's Law for vapor-liquid equilibrium:
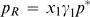
(11)in which γ_1_ is the activity coefficient and *p* ^*^ is the vapor pressure of pure water. The presence of protein is not considered to significantly affect *p_R_*, nor should it affect the water equilibration rates [7]. The expression for the water equilibration kinetics should provide the evolution of the droplet radius (or alternatively, of the corresponding volume, V) as a function of time, t. With that aim, Raoult's Law will be used to express the difference of vapor pressures in Equation 9 as a function of the precipitant molar fractions *x*_2_; in the reservoir, this value is assumed to remain equal to *x*_2*b*_, while in the droplet, it will increase as the droplet volume decreases. Accordingly, Equation 9 is rewritten as
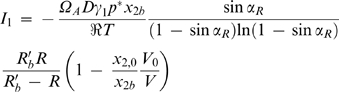
(12)where *x*_2,0_ and *V*_0_ are the initial precipitant molar fraction in the droplet and the initial droplet volume, respectively. The molar rate of water vapor leaving the droplet can also be expressed as a function of the droplet change of volume with time and of the molar volume of pure water (*V̅*_1_):
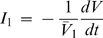
(13)Finally, knowing that the volume of droplet is given by
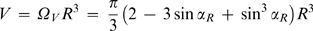
(14)and introducing the following dimensionless variable
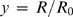
(15)Equation 12 becomes:
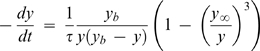
(16)where the constant *y_b_* corresponds to y evaluated at *R* = *R*′*_b_*, *y*_∞_ is the dimensionless radius of the droplet achieved at the equilibrium, when *dy*/*dt* = 0:
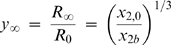
(17)and τ is a time constant defined by
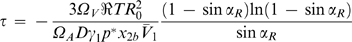
(18)It is now possible to obtain an expression relating the relative droplet radius (*y*) and the time elapsed since the beginning of the evaporation (*t*) by integrating Equation 16, subject to the initial condition *y*|*_t_*_ = 0_ = 1:
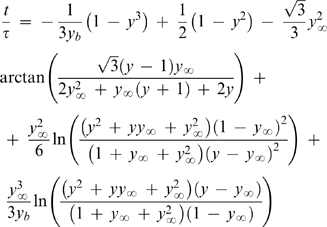
(19)The structure of this equation has similarities with the one obtained in the FM [3]. The main differences between the two models are, so far, the role of the droplet contact angle and the way vapor-liquid equilibrium (VLE) is expressed. The former difference vanishes in the cases where α*_R_* = 0°, and will not be discussed here. Concerning the VLE, while we propose Raoult's Law to relate the vapor pressure at the droplet surface and the droplet composition is given in terms of molar fractions (Equation 11), in the FM (as well as in the subsequent models SM and LM) that relationship is given as a function of the number of moles of water and salt in solution (*n*_1_ and *n*_2_, respectively), and of the vapor pressure lowering coefficient w:
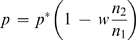
(20)The different formalisms adopted to describe the compositions in the droplet and in the reservoir also lead to differences in the resulting model equations. The practical consequences arising from each representation of the hanging drop method are illustrated in Figure 3. The differences between the two theoretical models are more evident in Figure 3B than in Figure 3A. This is partially because the authors of the experimental work presented in Figure 3A express their results in terms of the “percent completion” defined as [3], [12]:
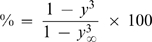
(21)This variable softens the differences between the predicted values of y in equilibrium (*y*_∞_). As we have previously defined (Equation 17), *y*_∞_ is a function of the 1/3 power of the relative molar fractions in the droplet and in the reservoir (dilution factor), while in the FM *y*_∞_ is a function of the molar volumes of water and precipitant, and of the respective number of moles in the droplet and in the reservoir [3]. The molar volume of MPD used in the simulations was of 118 cm^3^/mol [13]. Figure 3B illustrates that the relative volume in equilibrium predicted by the FM is significantly above the measured value of ∼0.5, which also corresponds to the value of *y* ^3^_∞_ expected from Equation 17.

In all cases considered in Figure 3, the theoretical curves correspond to faster evaporations than the measured ones. This is consistently observed using experimental data obtained under different conditions. One of the reasons for the gap between theory and practice might be related with the droplet contact angle, since all the theoretical simulations were performed assuming α*_R_* = 0°, when deviations to this value are referred to occur during the experiments [3], [7]. The original curve computed by Fowlis et al. takes into account the variations of the contact angle during the experiments and provided slightly slower evaporation rates than the FM curve plotted in Figure 3A [3]. Moreover, the geometrical simplification assuming *R_a_*≥*R_b_* and possibly, the overlooked role of mass transfer resistance within the droplet should also affect the quality of the predictions. In the following sections we shall address these two topics and, by doing it, improve the applicability of the proposed model.

### Long droplet-to-reservoir distances (*R_a_*≤*R_b_*)

**Figure 4:**
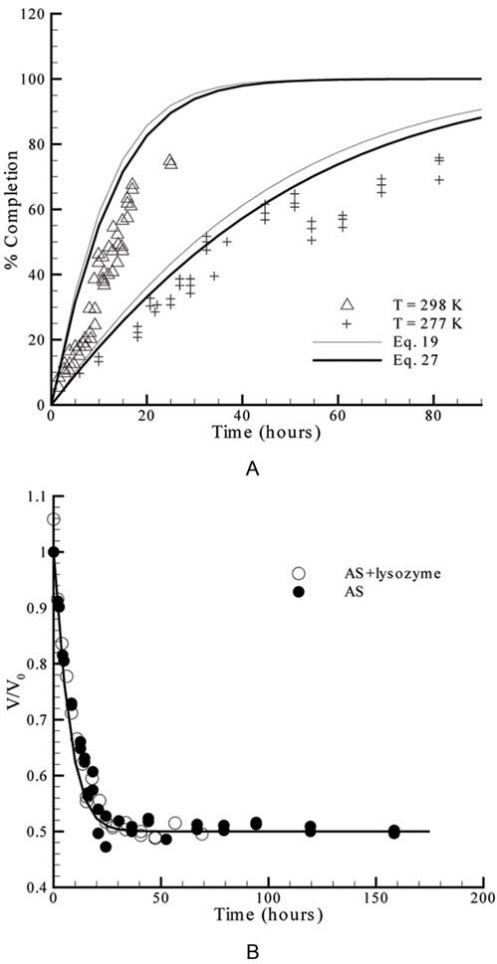
Validation of the predictions of Equation 27 against measured rates of water equilibration available in literature. A: Experimental sets #1 and #2 of Table 1 [3]. B: Experimental set #4 of Table 1 [7].

In the majority of the hanging drop apparatus, the droplet-to-reservoir distances are longer than the diameter of the reservoir, and so the presupposition *R_a_*≥*R_b_* would not be valid. The implications arising from this oversimplification are obviously greater for higher droplet-to-reservoir distances and narrower reservoirs. Recalling Figure 2, for *r*>*R_a_* the area for mass transfer represented by the surface of a spherical cap with radius r starts to be delimited by the walls of the reservoir, turning the angle α of the spherical cap a different function of r than for *r*<*R_a_* (Equation 4). Now, one finds from trigonometric transformations that:
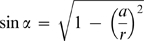
(22)The angle α provided by this equation when *r* = *R_a_* and *r* = *R_b_* will be called α*_a_* and α*_b_*, respectively. The geometrical shape of the vapor space should therefore be divided in two zones separated at *r* = *R_a_*. For *r* < *R_a_*, the vapor pressure profile is again obtained from Equation 5, subject to the boundary conditions *p*|*_r_*_ = *R*_ = *p_R_* and 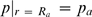, where *p_a_* is to be determined from the condition of continuous flux at the boundary (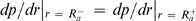). In the zone beyond *R_a_*, the continuity equation should be rewritten to account for the variation of α with r:
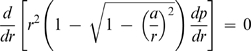
(23)Here the boundary conditions are 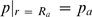 and 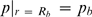. The ordinary differential equations (ODEs) 5 and 23 (and respective boundary conditions), together with the continuity condition at *r* = *R_a_*, constitute a boundary value problem representing the vapor pressure profile when *R_a_*≤*R_b_*. Replacing the solution of this problem in Equation 7, the following equation for *I*_1_ is obtained:
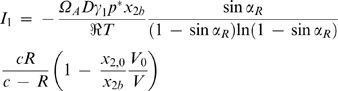
(24)with
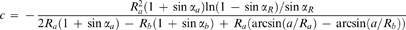
(25)The correspondence between Equations 12 and 24 is evident, with *R*′*_b_* of the former equation being replaced by c in the latter. Note that when *R_a_* = *R_b_* both equations are equivalent since it results from Equation 25 that *c* = *R*′*_b_*. Correspondingly, Equation 16 can be rewritten as
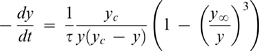
(26)where *y_c_* = *c*/*R*_0_, and can be solved to obtain the expression for the equilibration curves when *R_a_*≤*R_b_*:
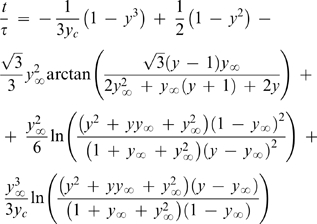
(27)This expression corresponds to Equation 19 after replacing *y_b_* by *y_c_*. Slower evaporation rates result from the introduced geometry corrections because the area for mass transfer is in this case smaller than when assuming *R_a_*≥*R_b_*. The greater *R_b_* relatively to *R_a_*, the bigger would be the differences to the preceding case. This is seen in Figure 4A by comparing the predicted profiles using Equations 19 and 27 (and the definitions of the respective parameters), for the experimental sets #1 and #2 of Table 1. Despite the small improvements, the water evaporation rates remain over-predicted by Equation 27. As it was pointed out in the discussion of Figure 3, variations of the droplet shape may explain the differences between the theoretical solution and the experimental results visible in Figure 4A. Likewise, in the previously considered case where MPD was used as precipitant (Figure 3B) a slight improvement of the theoretical profiles results from using Equation 27 instead of Equation 19 (data not shown). This is expectable since, as listed in Table 1 for experimental set #3, the reservoir radius (a) is close to the droplet-to-reservoir distance (b). In this case, the differences to the experimental results remain too high to be explained by variations on the droplet shape, only. It is believed that high mass transfer resistances within the droplet might explain the quantitative and qualitative differences obtained (see the following section). These resistances are expected to be more significant when using MPD as precipitant than with salts. The adequacy of Equation 27 in predicting water evaporation kinetics in the absence of significant liquid-phase resistances is confirmed in Figure 4B, which shows the results of a second experiment using ammonium sulfate as precipitant. In this experiment, the droplet́s shape did not change significantly from perfect hemispheres and a smaller droplet volume was used relatively to the experiment presented in Figure 4A (Table 1) [7]. As a result, the diffusion path within the droplet and the impact of the mass transfer resistance in the equilibration rates should also be smaller. The experimental results represented in Figure 4B also show that the presence of protein did not affect significantly the evaporation rates [7].

**Table 1:** Experimental details and physicochemical parameters of the water evaporation experiments used to validate the theoretical model.

Bibliographic Source	[3]		[7]		[5]	[7]	
**Experimental Set**	#1	#2	#3	#4	#5	#6	#7
**Crystallizing Agent**	Ammonium Sulfate (AS)		methyl-2,4-pentanediol (MPD)	AS (+lysozyme)	Sodium Chloride	MPD	PEG
***a*** ** (cm)**	0.85		0.80		0.68	0.80	
***b*** ** (cm)**	1.2		1.0		Variable	1.0	
***D*** ** (cm^2^/s)**	0.26	0.239	0.26†		0.26†	0.26†	0.239†
***p*** ^*^ (Torr)	23.769	6.101	17.5		21.07	17.5	5.7
***T*** ** (K)**	298	277	293		295.9	293	276
***V*** **_0_ (µl)**	25		32	8	24	8	16
***w*** ** (−)**	1.6259		1.403§	N/A	N/A	N/A	N/A
***x*** **_2_**_***b***_ **(−)**	0.0302	0.0314	0.0377	0.0321	0.0362	0.0377	0.0014
***x*** **_2,0_/** ***x*** **_2_**_***b***_ ** (−)**	0.5		0.5		0.5	0.5	
**γ_1_ (−)**	1.727§		1.779§	1.727§	1.752	1.779§	9.854§

§ Value estimated from freezing point depression measurements [7].

† The vapor diffusion coefficients reported at 298 K and 277 K [3] were not considered to change significantly for (i) 293 K and 295.9 K, and for (ii) 276 K, respectively.

**Figure 5:**
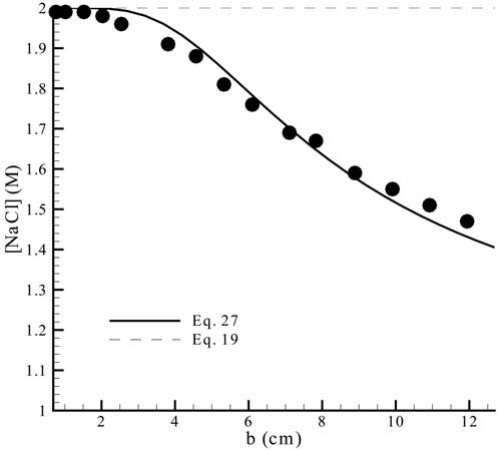
The measured influence of the droplet-to-reservoir distance on the average concentration of NaCl in the droplet after a 121 h evaporation period – experimental set #5 of Table 1 [5] – and the theoretical profiles expected from Equations 19 and 27 for the same set of conditions.

As demonstrated in the fundamental analysis presented so far, the applicability of theoretical models of the hanging drop method is in a good deal determined by the value of the droplet-to-reservoir distance (and its relation with the reservoir diameter). The same conclusion was drawn by measuring the dependence of water equilibration kinetics on the droplet-to-reservoir distance [5]. It was found that for small values of b the FM could be employed, while for large values the SM would be more suitable; at intermediate values of b a modified version of the FM and SM was proposed (LM), which introduces an adjustable parameter representing the effective surface area of the reservoir solution. The experimental results obtained by those authors are presented in Figure 5 in terms of the precipitant concentration in the droplet after a 121 h evaporation period, at different droplet-to-reservoir distances. The behavior expected by our model is also plotted in the same figure, after determining the y vs. b relationship from Equation 27 (and from the definitions of the respective parameters), and computing the instantaneous solute concentration in the droplet, C, as a function of y (*C* = *C*_0_/*y* ^3^, with the initial concentration *C*_0_ being 1.00 M). The same procedure was adopted, and applied to Equation 19, which predicts that the NaCl concentration should be independent of the droplet-to-reservoir distance after the considered evaporation period has elapsed. The agreement of the proposed model (Equation 27) with the collected data is remarkably good for low, intermediate, and large values of b (Figure 5), especially if one takes into account that no parameter was determined by curve-fitting to the evaporation rate results. In fact, for the simulations performed only the experimental parameters and physicochemical constants listed in Table 1 were used, which are generally available in the literature. Moreover, some degree of uncertainty resulting from possible variations of the droplet contact angle during the equilibration experiments is not taken into account on the simulations. The droplets were again considered to have a hemispherical shape (α*_R_* = 0°) that remains unchanged during the evaporation period. The four points of Figure 5 corresponding to higher values of b were obtained at slightly different temperature (297.2 K) and evaporation periods (120 h), and then corrected to the experimental conditions of the remaining points [5].

### Mass transfer resistance within the droplet

**Figure 6:**
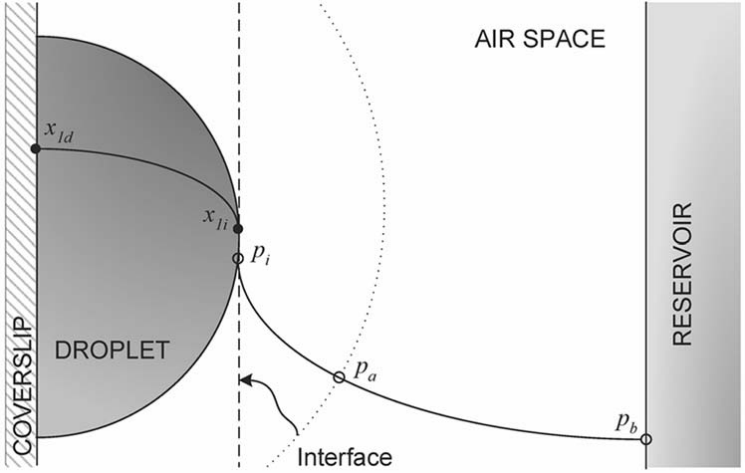
Representation of the water concentration profiles expressed in terms of molar fractions within the droplet, and of vapor pressures in the air space.

In the theoretical study done by Fowlis et al. on the several resistances that determine the evaporation rate during the hanging drop method, the diffusion of water molecules from the interior of the droplet to the surface was estimated to be of higher magnitude than the diffusion of vapor across the air space [3]. The same authors consider, moreover, that the convection motion under a gravitational field promotes homogeneous composition of the solution in the droplet and decreases the importance of the diffusion step in the liquid phase to a point that is no longer significant for the whole process. The results presented in Figures 4 and 5 seem to confirm that hypothesis whenever inorganic salts were used as precipitant; good predictions of the equilibration rates were obtained by considering that the rate limiting step was the vapor diffusion from the droplet to the reservoir. As previously referred, Figure 4B documents a contrasting case where the evaporation rates of droplets containing MPD were noticeably slower than the predictions. It is believed that the use of precipitants such as MPD or polyethylene glycol (PEG) may affect the rate of diffusion of water molecules and create a concentration gradient within the droplet (Figure 6). The additional diffusion resistance may explain the contradiction observed when solutes with a stronger vapor pressure lowering effect (like MPD relatively to ammonium sulfate) led to slower equilibration rates of water [7]. Incorporating the additional diffusion resistance in the theoretical model will be firstly done by changing the boundary conditions at the droplet surface. Equation 5, concerned with the vapor pressure profile for *r*<*R_a_*, is now subject to the boundary conditions *p*|*_r_*_ = *R*_ = *p_i_* and 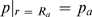, where *p_i_* is the effective interfacial vapor pressure. On the other hand, the interfacial vapor pressure is in equilibrium with the interfacial composition in the liquid phase represented in Figure 6 by the water molar fraction, *x*_1*i*_ (*x*_2*i*_, if one refers to the precipitant). Apart from the alteration in the boundary condition, the boundary value problem is solved as in the previous section to obtain a new equation for *I*_1_ that is analogous to Equation 24:
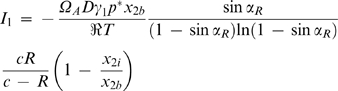
(28)The relationship between *x*_2*i*_ and the precipitant molar fraction in the bulk of the droplet, *x*_2*d*_, is given as a function of the mass transfer coefficient of water in solution, *k_c_*, using the following expression for the current *I*_1_:
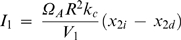
(29)


**Figure 7:**
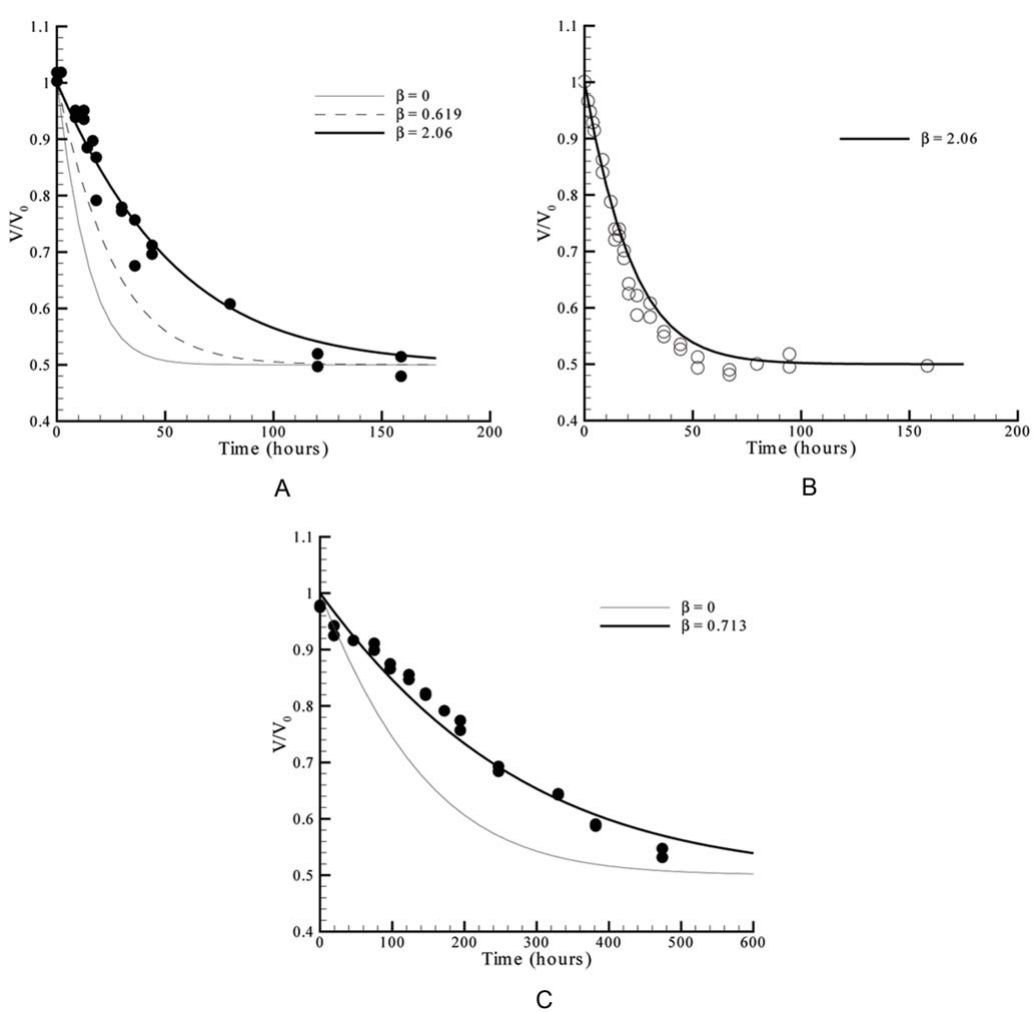
Validation of the predictions of Equation 32 against measured rates of water equilibration in the presence of significant diffusion resistance inside the droplet. A: Experimental set #3 of Table 1 [7]. B: Experimental set #6 of Table 1 [7]. C: Experimental set #7 of Table 1 [7].

The three definitions of *I*_1_ given by Equations 13, 28 and 29 can be combined to give the ODE of the variation of the droplet radius with time. Accordingly, after expressing *x*_2*d*_ as a function of the initial concentration in the droplet, *x*_2,0_, and using the dimensionless constants τ, *y*_∞_ and *y_c_*, one obtains
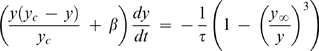
(30)where the introduced parameter β is defined as
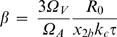
(31)and measures the weight of the mass transfer resistance within the droplet. Low mass transfer coefficients mean high resistances to diffusion and high values of β. The solution of Equation 31 (initial condition *y*|*_t_*_ = 0_ = 1) provides the most general form of the equilibration curve, contemplating liquid and vapor phase diffusion, and the cases where *R_a_*≤*R_b_*:
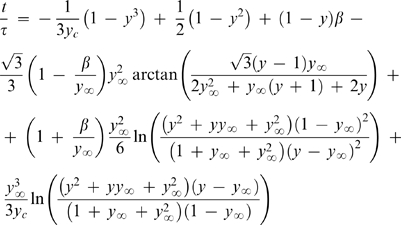
(32)It is now possible to predict the water evaporation rates for different levels of importance of the liquid phase resistance. This is done in Figure 7A for the already considered case of MPD used as precipitant, in Figure 7B for an analogous experiment with a different volume of the droplet (8 µl instead of 32 µl), and in Figure 7C where PEG was used as precipitant. As expected, the simulated equilibration rates represented in Figure 7A are progressively slower as the value of β increases, i.e., as lower mass transfer coefficients (*k_c_*) are considered in Equation 31. In this case, a good agreement with the experimental data was found for β = 2.06, which corresponds to *k_c_* = 1.5×10^−5^ cm/s. This value is close to a rough estimation of the mass transfer coefficient of 4×10^−5^ cm/s, obtained using a value of the liquid water diffusion coefficient of 10^−5^ cm^2^/s [14] and a diffusion path in the order of magnitude of the droplet radius (∼0.25 cm for a 32 µl hemispheral drop). The value of *k_c_* obtained from the results shown in Figure 7A was used to predict the equilibration rates of an analogous experiment with smaller droplet radius (Figure 7B); a correction factor of (32/8)^1/3^ was applied to that value to account for the new diffusion path in the smaller drop, so that in the simulation of Figure 7B, *k_c_* = 2.38×10^−5^ cm/s. As it arises from the definition of β, the same value of 2.06 is obtained for this parameter after the droplet radius correction. Accordingly, the equilibration rates of the 8 µl droplets were simulated and the predicted profiles are again in good agreement with the experimental data (Figure 7B). This is a good indication that mass transfer coefficients determined for a given system can be later used, after the corrections to the diffusion path, and eventually temperature. Figure 7C is concerned with another situation where the used precipitant (PEG) may have led to significant liquid phase resistances. The results are qualitatively and quantitatively well described using β = 0.713, which corresponds to a mass transfer coefficient of *k_c_* = 1.0×10^−4^ cm/s. The reasons why this coefficient is higher than those obtained with solutions of MPD are related with different diffusion properties of each solution, as well as with the different diffusion paths and temperature in each of the cases. The VLE data determined for PEG from the measurement of the freezing point depression [7] has some scatter that may also have affected the quality of the predictions represented in Figure 7C.

### Applicability of the model and future work

Once proposed and validated a solvent equilibration rate model, the next step for rational protein crystallization should be the combination of this model with the protein solubility information in order to know the evolution of supersaturation in vapor diffusion techniques. At any time during a hanging drop crystallization experiment, the volume of the droplet can be calculated from the dimensionless droplet radius y obtained from Equation 32, for the set of constants τ, β, *y_c_* and *y*_∞_ that are characteristic of the system. Then, the instantaneous concentration of precipitant can be computed from the droplet volume. Having the protein solubility curve measured at the pH and temperature conditions under study and the calculated concentration of precipitant, the corresponding protein solubility is available. Finally, the supersaturation can be calculated, using the updated values of protein concentration and solubility. The information conventionally provided in phase diagrams of the nucleation region in relation to the solubility curve [2], [15] can now be complemented with the kinetic characterization of the process between the initial and final stages of a crystallization experiment.

The goal is to recognize the kind of supersaturation profiles that lead to high quality protein crystals and to know how to achieve the corresponding evaporation kinetic profiles. At this point, a distinction should be made between purely “kinetic” parameters, which affect solely the water equilibration rates, and those that also affect the thermodynamics of the solution. In the first category are included the geometry of the crystallization chamber, the droplet-to-reservoir distance, and the droplet volume, while for the second category the examples are temperature, pH, and the precipitant type and concentration. Purely “kinetic” parameters are therefore appropriated for fine-tuning of the crystallization conditions, and their effect can be promptly computed using Equation 32. When the second-type parameters are changed, besides their effect on the evaporation kinetics (Equation 32), the solution thermodynamics is also altered according to the solubility curve of the system. We envisage that future work will address the incorporation of nucleation and crystal growth models on the full characterization of the crystallization technique. This is important, but not straightforward since several parameters need to be studied. For example, depending on the hydrophobicity of the coverslip material, different evaporation kinetics are expected (due to different contact angles and shapes of the droplet) but also different nucleation kinetics will occur [16], [17].

The applicability of Equation 32 can also be extended in the future to vapor diffusion techniques in the presence of oils [18]. In fact, considering the apparatus of Figure 2, a layer of oil can be applied upon the solution in the reservoir or covering the droplet. The practical consequence of this procedure is to lower the liquid-vapor mass transfer coefficient and to decrease the evaporation rates. If the oil is totally water-impermeable, evaporation will be suppressed, leading to a microbatch crystallization experiment. Other vapor-diffusion methods such as sitting drop share the same principles here illustrated for the hanging drop method, although they are subject to different, and generally more complex, geometrical constraints that depend on the crystallization chamber design, employment of micro-bridges or glass rods as droplet holders, etc. In future, it is of interest to investigate the differences in the water equilibration rates of the different possible arrangements by measuring the corresponding evaporation kinetics and, when possible, provide the theoretical basis of the measured data. The validation results here reported for the hanging drop method are a good indication that a priori screening of crystallization conditions is a goal not too far to be accomplished if protein solubility curves under different conditions are experimentally determined.

### Conclusions

A new equilibration rate model was presented describing the hanging drop method for protein crystallization. The presentation of the model was progressively made with increasing degrees of complexity as a consequence of the model assumptions that were being considered. This also allowed emphasizing the differences between the present theory and existing models, namely the distinct ways of characterizing the vapor-liquid equilibrium, the geometrical assumptions involved in the different models, and the role allocated to water diffusion within the droplet. Several measurements of water equilibration rates using different precipitants at the different experimental conditions were taken from literature and compared with the predictions of the proposed model. The experimental curves described the variation of the droplet volume (or alternatively, the precipitant concentration in the droplet) with the evaporation time, and with the droplet-to-reservoir distance, at a fixed evaporation time. Good validation results were obtained in all cases. In the performed simulations, only the experimental parameters and physicochemical constants reported in the analyzed literature data were used. Precipitants which are expected to significantly increase the water diffusion resistance within the droplet were indentified. The clearest example was MPD, for which water mass transfer coefficients were possible to be estimated for the liquid phase. The obtained coefficient was then used to successfully predict the water equilibration rates in the presence of MPD, at different conditions. The proposed model will be used to predict the water evaporation rates in a variety of different conditions used in the hanging-drop vapor diffusion technique for protein crystallization. In fact, this model is a consistent step for a rational protein crystallization experimental set up.
